# Dissociation of Tissue Destruction and Bacterial Expansion during Bubonic Plague

**DOI:** 10.1371/journal.ppat.1005222

**Published:** 2015-10-20

**Authors:** Françoise Guinet, Patrick Avé, Sofia Filali, Christèle Huon, Cyril Savin, Michel Huerre, Laurence Fiette, Elisabeth Carniel

**Affiliations:** 1 Unité de Recherche *Yersinia*, Institut Pasteur, Paris, France; 2 Unité d’Histopathologie Humaine et Modèles Animaux, Institut Pasteur, Paris, France; 3 Unité de Recherche et d’Expertise d’Histotechnologie et Pathologie, Institut Pasteur, Paris, France; University of Michigan Medical School, UNITED STATES

## Abstract

Activation and/or recruitment of the host plasmin, a fibrinolytic enzyme also active on extracellular matrix components, is a common invasive strategy of bacterial pathogens. *Yersinia pestis*, the bubonic plague agent, expresses the multifunctional surface protease Pla, which activates plasmin and inactivates fibrinolysis inhibitors. Pla is encoded by the pPla plasmid. Following intradermal inoculation, *Y*. *pestis* has the capacity to multiply in and cause destruction of the lymph node (LN) draining the entry site. The closely related, pPla-negative, *Y*. *pseudotuberculosis* species lacks this capacity. We hypothesized that tissue damage and bacterial multiplication occurring in the LN during bubonic plague were linked and both driven by pPla. Using a set of pPla-positive and pPla-negative *Y*. *pestis* and *Y*. *pseudotuberculosis* strains in a mouse model of intradermal injection, we found that pPla is not required for bacterial translocation to the LN. We also observed that a pPla-cured *Y*. *pestis* caused the same extensive histological lesions as the wild type strain. Furthermore, the *Y*. *pseudotuberculosis* histological pattern, characterized by infectious foci limited by inflammatory cell infiltrates with normal tissue density and follicular organization, was unchanged after introduction of pPla. However, the presence of pPla enabled *Y*. *pseudotuberculosis* to increase its bacterial load up to that of *Y*. *pestis*. Similarly, lack of pPla strongly reduced *Y*. *pestis* titers in LNs of infected mice. This pPla-mediated enhancing effect on bacterial load was directly dependent on the proteolytic activity of Pla. Immunohistochemistry of Pla-negative *Y*. *pestis*-infected LNs revealed extensive bacterial lysis, unlike the numerous, apparently intact, microorganisms seen in wild type *Y*. *pestis*-infected preparations. Therefore, our study demonstrates that tissue destruction and bacterial survival/multiplication are dissociated in the bubo and that the primary action of Pla is to protect bacteria from destruction rather than to alter the tissue environment to favor *Y*. *pestis* propagation in the host.

## Author Summary

The hallmark of bubonic plague, a disease that ravaged Medieval Europe and is still prevalent in several countries, is the bubo, a highly inflammatory and painful lymph node, which is characterized by high concentrations of bacteria within a severely damaged organ. *Yersinia pestis*, the causative agent, expresses a surface protease, Pla, critical to the development of bubonic plague. This multitarget protease has the potential to activate the fibrinolytic pathway and to promote destruction of extracellular protein networks within tissues. Hence, it was expected that Pla was responsible for the tissue destructions of the bubo, and consequently, for bacterial propagation and virulence. However, we found, using various engineered *Yersinia* strains in a mouse model of bubonic plague, that Pla proteolytic activity was dispensable for lymph node alteration, but was required to achieve high bacterial loads in the organ. Further analysis showed that Pla is essential for preventing the bacteria from being destroyed in the host. Therefore, the role of Pla as a virulence factor is to protect *Y*. *pestis* survival and integrity in the host, rather than to assist its spread through tissue destruction.

## Introduction

Plague killed millions of humans during pandemics of the past and is still entrenched in regions of Asia, Africa and the Americas [1,2]. The last decades have witnessed resurgences and geographical extensions of the disease, leading WHO to categorize it as a re-emerging health problem [3,4], and there are concerns that future climatic changes might further increase the occurrence of plague outbreaks in existing or new foci [2].

Bubonic plague is the most frequent form of the disease and results from intradermal injection by an infected flea of the Gram-negative bacterium *Yersinia pestis* [5,6]. Bacteria proceed then, via lymphatic draining, to the proximal lymph node and expand in this organ to high numbers of widespread and infiltrating extracellular organisms [7–11]. At this stage, the swollen and highly painful draining lymph node (dLN) is referred to as a “bubo”. Without treatment, bubonic plague most often progresses to fatal septicemia [12,13]. The 50% lethal dose (LD_50_) of *Y*. *pestis* in mice is <10 and ~20 colony forming units (cfu) by the subcutaneous (sc) and intradermal (id) routes, respectively [6,14–16].

*Y*. *pestis* is a clonal species recently emerged from the foodborne enteropathogen *Y*. *pseudotuberculosis* [17], which causes self-limiting gastrointestinal diseases in humans [18,19] and has an LD_50_ in mice of 10^5^−10^7^ cfu following oral or sc inoculation [20,21]. Therefore, although the two species are genetically nearly identical [22], they display dramatically different pathogenic potentials. In a previous work, we used the *Y*. *pestis*/*Y*. *pseudotuberculosis* pair to explore the pathophysiology of bubonic plague [16]. Comparison of the diseases induced upon id injection of the two species showed that the dermal portal of entry newly acquired by *Y*. *pestis* is not the key to its increased virulence. The study also revealed specific histology features in the dLN within 2 days of infection. In buboes, plague was characterized by high bacterial loads, poorly contained bacterial infiltrates and widespread tissue destruction; in *Y*. *pseudotuberculosis*-infected dLNs, bacteria formed dense colonies enclosed in a focal and organized inflammatory reaction while the overall architecture of the dLN was preserved.

At this stage, it was not clear if and how the characteristics of the mature bubo were connected in a pathophysiological pathway. The severe tissue damage might have been a consequence of the high bacterial burdens, through the action of bacterial or immunological components, and conversely, destructions of the lymph node parenchyma, by removing tissue barriers, could have been a facilitating factor for the free diffusion and expansion of *Y*. *pestis* within the organ. The *Y*. *pestis* pPla plasmid is one of the few genetic determinants that differentiate the plague bacillus from its *Y*. *pseudotuberculosis* ancestor [22]. Since this plasmid has been shown to participate in bacterial dissemination *in vivo* and to play a major role in *Y*. *pestis* virulence [15,23–25], it was a potentially useful tool to analyze the relationships between the various features that specify a *Y*. *pestis*-infected dLN and to relate these features to virulence. pPla encodes Pla, a protease of the omptin family [26,27], which cleaves mammalian plasminogen into active fibrinolytic plasmin and inactivates several inhibitors of the fibrinolytic pathway [28]. Pla also degrades antibacterial factors [15,23,29], manipulates *in vivo* the pro-inflammatory response through interference with the FasL/caspase-3 pathway [30], and confers adherence to various cell lines and extracellular matrix components, either directly [31–36], or through processing of the YapE adhesin [37]. The *pla* gene encoding Pla is abundantly expressed in the dLN of *Y*. *pestis*-infected mice [38]. In heterologous expression systems, the proteolytic action of Pla at the bacterial surface is hampered by the O-antigen (O-Ag) fraction of the outer membrane lipopolysaccharide [39]. However, Pla is fully active in *Y*. *pestis* because genes encoding the O-Ag synthesis enzymes are naturally non-functional in this species [40].

The aim of this work was to use *Y*. *pestis* and *Y*. *pseudotuberculosis* strains differentially expressing Pla to analyze three defining characteristics of the bubo: infiltrating and diffuse pattern of bacterial invasion, extensive and disrupting tissue damage, and elevated bacterial titers. This approach helped us dissect the bubonic plague complex phenotype, uncovering new insights into the causal links between the various manifestations of the disease and their relation to outcome.

## Materials and Methods

### Animal ethics

Animals were housed in the Institut Pasteur BSL3 animal facility accredited by the French Ministry of Agriculture to perform experiments on live mice (accreditation B 75 15–01), in compliance with French and European regulations on care and protection of Laboratory Animals (EC Directive 86/609, French Law 2001–486 issued on June 6, 2001). Protocols were approved by the Institut Pasteur Veterinary Staff and performed in compliance with the NIH Animal Welfare Insurance #A5476-01 issued on 02/07/2007.

### Bacteria and plasmids

Bacterial strains and plasmids used in this study are listed in Table 1. Bacteria were grown using Luria-Bertani medium (LB), supplemented (*Yersinia*) or not (*Escherichia coli*) with 0.002% (w/v) hemin (LBH), at 28°C and 37°C, respectively. Selection for trimethoprim resistance was done using Müller-Hinton medium. Chloramphenicol (25 μg.ml^-1^), kanamycin (Km: 30 μg.ml^-1^) or trimethoprim (Tmp; 100 mg.ml^-1^) was added to the media as necessary. All experiments involving *Yersinia* strains were performed in a BSL3 laboratory.

**Table 1:** Plasmids and bacterial strains.

Plasmid or strain	Relevant features	Source
*Plasmids*		
pPlaTmp	pPla plasmid of *Y*. *pestis* CO92 containing a dfr cassette; Tmp^R^	[21]
pPlaD206A	pPlaTmp with a nucleotide mutation at position 206 that abrogates the proteolytic activity of Pla	This study
pGP704N-Km	Suicide vector, Amp^R^, Km^R^	[43]
pPlaKm	pPlaTmp plasmid containing a kanamycin cassette inserted into the *dfr* gene; Km^R^, Tmp^S^.	This study
pKOBEG-sacB	*repA cat araC pBAD exo bet gam sacB*, Cm^R^	[42]
***Y*. *pestis***		
Yp.wt	Wild type *Y*. *pestis* strain 6/69, biotype Orientalis	[50]
Yp(ΔPla)	6/69 cured of the pPla plasmid	[43]
Yp(PlaD206A)	pPlaD206A introduced into Yp(ΔPla) by electroporation	This study
Yp(pKOBEG-sacB) (pPlaTmp)	*Y*. *pestis* CO92 strain (biotype Orientalis) containing the pKOBEG-*sacB* and the pPlaTmp plasmid	[21]
Yp(PlaD206A)(pPlaKm)	Yp(PlaD206A) complemented with pPlaKm to restore the proteolytic activity of Pla	This study
***Y*. *pseudotuberculosis***		
Yptb*	*Y*. *pseudotuberculosis* IP32953 in which O-antigen expression has been abrogated by deletion of the *gmd* and *fcl* loci.	[21]
Yptb*(Pla)	pPlaTmp introduced into Yptb* by electroporation	[21]

### Mutagenesis of pla

Single nucleotide replacement in *pla* was performed by PCR amplification of the whole 9.6 kb pPlaTmp plasmid from strain Yptb*(Pla) (Table 1), using the divergent non-overlapping primers 896 and 897 harboring the required mutation (S1 Table) and a high-fidelity DNA Polymerase (Phusion, NewEnglandBiolab) [41]. The 80 μl reaction mix, containing 0.125 mM dNTP, 0.1875 μM of primers and 0.5 μl of Taq Polymerase, was incubated for 5 min at 98°C and subjected to 30 cycles of denaturation (30 s at 98°C), annealing (30 s at 60°C), and extension (3 min 30 s at 72°C), and a final cycle of extension (7 min at 72°C). The resulting PCR product was digested with *Dpn*I to cleave the methylated template and subjected to electrophoresis. The band with the expected size was excised from the gel, extracted using the QIAquick Gel Extraction Kit (Qiagen), and introduced into *E*. *coli* DH5α by transformation. Recombinant clones were selected on Tmp plates and plasmids were extracted using the Plasmid maxi kit (Qiagen). Sequencing of the entire replicon, using the primers listed in S1 Table showed that it was 100% identical at the nucleotide level to the pPla template, except at position 7,341 in *pla* where the A was replaced by a C at the second position of the codon, leading to the expected replacement of an aspartic amino acid by an alanine at position 206 of the Pla protein sequence. The recombinant plasmid, designated pPlaD206A, was introduced by electroporation into Yp(∆Pla). Recombinant *Y*. *pestis*, designated Yp(PlaD206A) (Table 1), were selected on Tmp agar plates and the presence of the Tmp cassette was verified by PCR with primer pair 260B/C (S1 Table). To confirm the presence of the recombinant pPlaD206A plasmid, the PCR product encompassing this region (primers 839A/B (S1 Table)) was digested with *Blp*I, which only cleaves the A/C mutated site.

### Complementation of Yp(PlaD206A) with a functional pla gene

In order to obtain a pPla plasmid labeled with an antibiotic cassette other than Tmp (already present on pPlaD206A), the *dfr* locus of the pPlaTmp plasmid was replaced by a Km cassette using the short flanking homologous regions (SFH)-PCR procedure [42]. Briefly, the Km locus was PCR amplified from the pGP704N-Km template [43] with primer pair 954A/B (S1 Table) that encompass the extremities of the Km cassette and 50 bp of the *dfr* gene. This PCR product was introduced into Yp(pKOBEG-sacB)(pPlaTmp) [21] by electroporation. Km^R^ colonies were tested for correct allelic exchange between the PCR product and the target site by PCR with primers 932/933 (S1 Table) and 260B/C [21], located at each end of the *kan* cassette and outside the *dfr* gene, respectively. All colonies contained both pPlaTmp and pPlaKm. To obtain *Y*. *pestis* clones containing pPlaKm, but devoid of pPlaTmp, the plasmids were extracted with the QuickLyse Miniprep kit (Qiagen) and introduced by electroporation into Yp(ΔPla). Km^R^ clones, designated Yp(pPlaKm) (Table 1) were selected. The presence of the pPlaKm and the absence of pPlaTmp were confirmed by PCR with primer pairs 932/933 and 260B/C. Transfer of pPlaKm to Yp(PlaD206A) after plasmid extraction and electroporation resulted in the generation of Yp(PlaD206A)(pPlaKm) (Table 1). The presence of both the wild type and the mutated versions of *pla* was verified by amplification of the region encompassing the A -> C replacement on *pla* with primers 839A/B (S1 Table), followed by digestion with *Blp*I. The presence of three bands (one corresponding to the uncut wild type version of *pla*, and the other two to the fragments generated by the *Blp*I cleavage), confirmed the presence of the two forms of pPla in the strain.

### Plasminogen-activation assay

The assay was performed as described [21,44], with slight modifications: bacteria were incubated at 37°C in Ca/Mg-free phosphate buffer containing 4 μg human Glu-plasminogen (American Diagnostica) and 30 μl of a 3 mM solution (in H_2_0) of the chromogenic plasmin substrate S-2251 (Chromogenix) in a total volume of 200 μl. Breakdown of the chromogenic substrate was monitored by serial measurements of absorbance at 405 nm (A_405_) using a microtiter-plate reader. Experiments were performed in duplicate and at least twice.

### Mouse infection

Eight-week old female OF1 mice (Charles River, France) were anesthetized by intraperitoneal injection of 10 mg.kg^-1^ Xylazine (Rompun 2%, Bayer, Germany) + 100 mg.kg^-1^ Ketamine (Imalgène 1000, Merial, France) and infected as described [16,45]. Briefly, bacteria grown overnight on LBH agar medium were adjusted to the desired concentration in saline, based on A_600_ measurement, and 10 μl (5,000 cfu) were injected id into the mouse ear pinna. Cfu counts were verified by plating on LBH. Control mice received the same volume of saline without bacteria. Mice were followed up for 21 days for survival. For cfu enumeration or histology examination of the LN, infected mice were euthanized by cervical dislocation at 48h post-infection (pi), unless otherwise specified, and the ipsilateral superficial parotid LN [46], which drains the injection site [16], was harvested.

### Histology and immunohistochemistry

LNs were first collected in a Zinc-based preservative [47,48] and then embedded in low melting-point paraffin (Poly(ethylenglycol) diesterate, Aldrich). Four μm sections were stained with standard hematoxylin-eosin (HE) staining [49]. For immunohistochemistry, sections were treated for endogenous peroxidase activity by incubation for 20 min in 0.3% (v/v) H_2_O_2_, and for 20 min in normal serum from the appropriate animal host (dilution 1:10 in PBS (pH 7.4) containing 1% (w/v) milk powder) prior to incubation for 1 h with one of the following antibodies: rabbit polyclonal antiserum against the *Y*. *pestis* F1 Ag or the *Y*. *pseudotuberculosis* serotype I O-Ag (produced by the French Reference Center for *Yersinia*, Institut Pasteur), or a rat anti-mouse CD45R (B220 clone, Caltag). After three washes in PBS-1% (w/v) milk powder, LN sections were incubated for 1 h with the following secondary antibodies or reagent (Dako): EnVision+System HRP-labeled anti-rabbit (undiluted), streptavidin-peroxidase conjugate (diluted 1:600), or rat-specific biotinylated Ig (diluted 1:400) followed by streptavidin-peroxidase conjugate. Bound peroxidase activity was detected using 3-amino-9-ethylcarbazole (AEC) substrate (Sigma). Tissues were counterstained with Harris’ hematoxylin. Sections were photographed with a DMX1200 or DS-Fi1 Nikon Camera connected to an E800 or E600 Nikon microscope equipped with 2× to 100× Plan Apochromat objectives, or scanned with a MiraxScan Z1 from Zeiss.

### Statistical analyses

Statistical analyses were performed with the Prism version 5 for Mac software (GraphPad Software, San Diego California), using the Student’s *t* test or Mann-Whitney *U* test, depending on the distribution of the data, to compare bacterial loads in organs and the log-rank test to compare mortality rates.

Clustering analysis of the LN lesional patterns: histology sections were scored according to previously described criteria of histology lesions in the lymph node [16]. Each sample was scored as « 0 » or « 1 » for each of the criteria related to tissue damage and inflammatory reaction, so that a « 0 / 1 » table gathering the lesional patterns of all samples was created. The table was computed by the BioNumerics software, version 6.6 (Applied Maths, Kortrijk, Belgium), using the Minimum Spanning Tree approach to analyze and display the similarity of the patterns.

## Results

### *Y*. *pestis* infiltrating pattern of progression in the bubo does not require pPla

**Fig 1:**
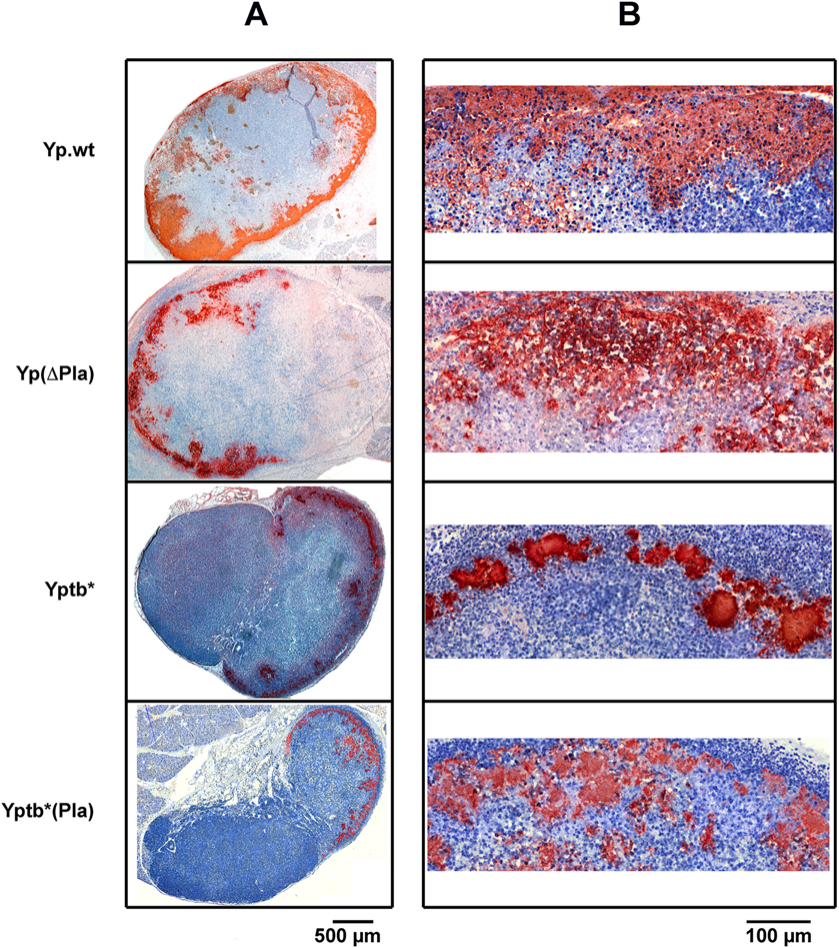
Distribution and infiltration pattern of bacteria in dLNs infected with *Y*. *pestis* or *Y*. *pseudotuberculosis* strains containing or not the pPla plasmid. Mice were infected id in the ear with 5x10^3^ cfu of each strain. At 48 h post infection mice were sacrificed and the ipsilateral superficial parotid LN was collected. LN sections were immunostained with an anti-*Y*. *pestis* (first two rows) or -*Y*. *pseudotuberculosis* (last two rows) antiserum. Bacteria have an orange-brown coloration, and the tissues are counterstained with hematoxylin (blue). (A) and (B) panels represent low- and high-magnification pictures, as indicated by the corresponding scale bars. Strains are denoted as in Table 1. The figure shows typical aspects observed in a panel of 68 examined LNs (20 infected with Yp.wt, 26 with Yp(ΔPla), 11 with Yptb* and 11 with Yptb*(Pla)).

Following injection of *Y*. *pestis* into the dermis, bacteria disseminate to the dLN. They first settle and spread in the subcapsular sinus, from which multifocal bacterial extensions subsequently penetrate into the cortex [11,16]. In contrast, the closely related species *Y*. *pseudotuberculosis* forms in the dLN discrete peripheral clumps separated from the parenchyma by an inflammatory cell mantle [16]. To determine whether the *Y*. *pestis*-specific plasmid pPla was responsible for the infiltrating behavior of *Y*. *pestis*, we used a set of four strains: (i) wild type *Y*. *pestis* (Yp.wt), (ii) its derivative cured of the *pla*-bearing plasmid pPla (Yp(ΔPla)), (iii) a *Y*. *pseudotuberculosis* strain in which O-Ag production has been abrogated (Yptb*) [21], and (iv) its derivative in which pPla was introduced (Yptb*(Pla), [21] (Table 1). The use of an O-Ag-deprived strain of *Y*. *pseudotuberculosis* was necessary because the activity of Pla is inhibited in the presence of the lipopolysaccharide side chains [39]. We previously showed that the plasminogen activator activity of the recombinant Yptb*(Pla) strain is similar to that of Yp.wt [21]. Each of the four strains was inoculated in the mouse ear pinna at a dose of 5,000 cfu. This loading dose was found in an earlier work to produce well-developed lymphadenites exhibiting distinct features of *Y*. *pestis* versus *Y*. *pseudotuberculosis* infection [16]. Two days after infection, the animals were euthanized and the ipsilateral superficial parotid dLN was taken for microscopic examination. Immunostaining of dLNs infected with either Yp.wt or Yp(∆Pla) evidenced diffuse and infiltrating bacterial projections towards the LN center in both cases (Fig 1A). The similar pattern of bacterial infiltration between pPla-positive and -negative *Y*. *pestis* was confirmed at higher magnification (Fig 1B). In contrast to *Y*. *pestis*, Yptb* and Yptb*(Pla) assumed a less diffuse spatial distribution, forming compact bacterial patches located at the dLN periphery (Fig 1A). However, at higher magnification, Pla-expressing *Y*. *pseudotuberculosis* bacteria were often found to form less densely packed colonies than Yptb* (Fig 1B), suggesting that pPla might slightly increase the diffusing potential of *Y*. *pseudotuberculosis*.

Therefore, although pPla may participate in the infiltrating pattern that typifies a bubo, its role appears to be minor. This plasmid is not responsible for the difference in bacterial containment observed between *Y*. *pestis* and *Y*. *pseudotuberculosis*, and is not a requisite for *Y*. *pestis* infiltration of the dLN.

### Destructive lesions in the bubo are not caused by pPla

**Fig 2:**
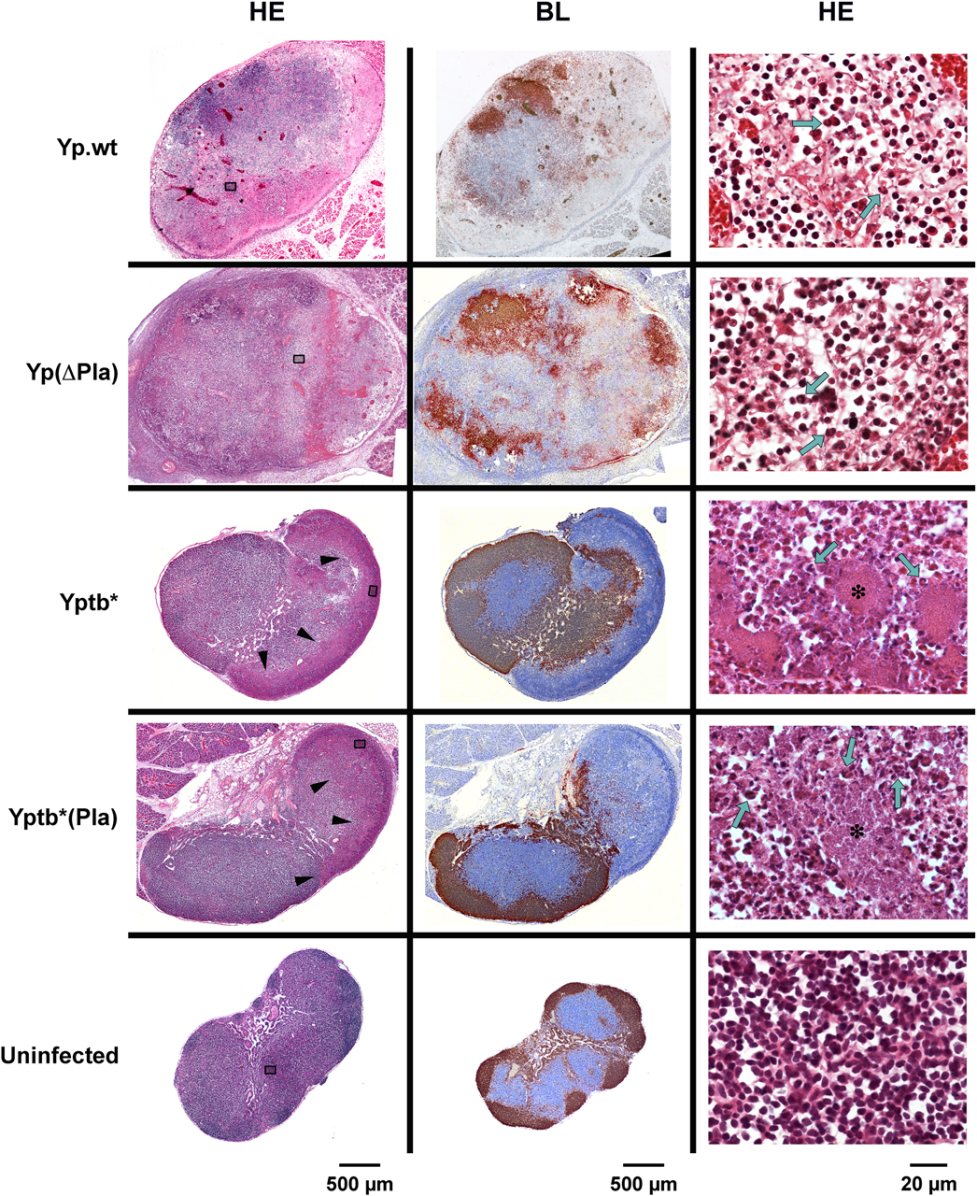
Histology of dLNs infected with *Y*. *pestis* or *Y*. *pseudotuberculosis* strains containing or not the pPla plasmid. Mice were infected id in the ear with 5x10^3^ cfu of each strain and dLNs were collected 48h later. An uninfected LN is shown for comparison. HE: hematoxylin-eosin staining; BL: immunostaining of B lymphocytes (brown), and counterstaining with hematoxylin (blue). In Yp.wt- and Yp(ΔPla)-infected nodes (first two rows): areas of lymphoid tissue depletion and necrosis appear on low-magnification HE pictures (left column) as zones of lower staining intensity or as irregular pink areas; at higher magnification (right column), dissociation of the lymphoid tissue, deposition of eosinophilic fibrillar material, and the presence of necrotic cells and cell debris characterize the necrotic process; typical PMNs harboring horseshoe-shape nuclei are visible (green arrows). In Yptb^*^ and Yptb^*^(Pla)-infected nodes (third and fourth rows): on low-magnification HE pictures, arrowheads indicate the limits of the inflammatory infiltration clearly delineated from the rest of the organ; higher magnifications show that the inflammatory infiltration is essentially made of PMNs (green arrows), which come in close contact with the bacterial foci (stars). In uninfected LNs (last row), the lymphoid tissue density and follicular organization (B cells at the periphery) are normal. Strains are denoted as in Table 1. Sections of infected dLNs shown here are from the same dLNs as in Fig 1. The position of the fields shown in the right column is marked by grey rectangles in the corresponding low-magnification HE sections.

We previously showed that on day 2 pi, the dLNs of mice infected id with *Y*. *pestis* exhibited destructive lesions leading to alterations of the tissue density and a breakdown of the functional organization of the organ, while dLNs of mice infected with *Y*. *pseudotuberculosis* displayed peripheral abscesses but kept an otherwise normal architecture [16]. To estimate the contribution of pPla to the destruction of the LN architecture, mice were infected with Yp.wt, Yp(ΔPla), Yptb* and Yptb*(Pla) and LN histopathology was examined at 48h pi. Hematoxylin-eosin (HE) staining of the dLNs infected with either Yp.wt or Yp(ΔPla) revealed comparable heterogeneous tissue structures with zones of tissue depletion and necrosis (Fig 2, HE). Disruption of the LN follicular organization by both pPla-positive and -negative *Y*. *pestis* was also seen on sections immunostained to reveal B lymphocytes: these cells no longer occupied the outermost regions of the organ, as in normal LNs. They composed instead fragmented islets that were not restricted to the periphery (Fig 2, BL). LNs infected with Yptb* or Yptb*(Pla) (Fig 2) displayed in both cases preserved tissue density (Fig 2, HE) and the follicular architecture was also conserved, as B cells were located at the organ periphery or homogeneously forced inward by an organized and contained inflammation (Fig 2, BL). A grouping analysis of the histology lesions confirmed that the lesional profiles clustered according to the infecting species, and not to the presence of Pla (S1 Fig).

These findings thus show that histological features characteristic of the plague bubo, i.e. disappearance of the organ functional architecture, destructive alterations of the tissue, and lack of an organized innate cell response [10,11,16], are specific for the plague bacillus, but independent of pPla. Therefore, pPla is neither required nor sufficient for the destruction of the LN structure that characterizes a *Y*. *pestis* infection.

### dLN bacterial titers are determined by Pla catalytic activity

**Fig 3:**
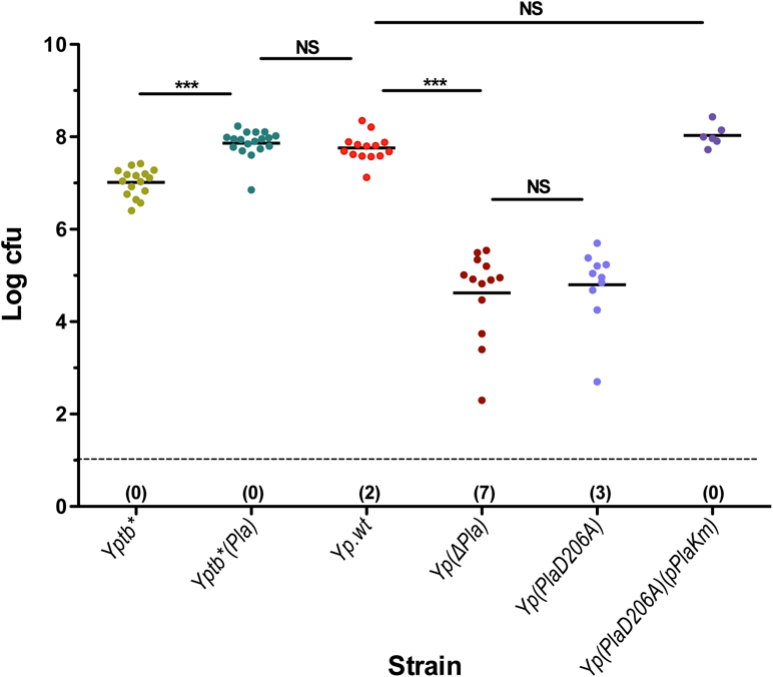
dLN colonization by *Yersinia* strains differentially expressing the proteolytic activity of Pla. Mice were infected id in the ear with 5x10^3^ cfu of each strain. At 48 h pi mice were sacrificed and colonization of the ipsilateral superficial parotid LN was determined. Each dot represents an individual animal. Black bars correspond to the mean number of cfu in colonized LNs. The dashed line indicates the limit of detection. In parenthesis below the dashed line is indicated for each group the number of LNs with no detectable live bacteria. Mean numbers of cfu in colonized LNs were compared using t-test, as data were normally distributed in all groups. NS: *P* > 0.05; ***: *P* ≤ 0.0001. Strains are denoted as in Table 1.

Overwhelming bacterial loads in mature buboes represent another distinctive feature of *Y*. *pestis* infections [11,12,16]. This characteristic has been linked to the presence of pPla by histological observations [15,24], but no quantification of the effect of pPla on bubo bacterial loads has ever been performed. To quantitatively assess the impact of pPla on the bacterial ability to multiply in the dLN, 5,000 cfu of Yp.wt, Yp(∆Pla), Yptb* and Yptb*(Pla) were inoculated id and cfu enumerations in the dLN were carried out on day 2 pi. As shown in Fig 3, loss of pPla by *Y*. *pestis* resulted in a reduced proportion of infected dLNs (87.5% for Yp.wt versus 65% for Yp(∆Pla)), and in dLNs that were infected the amount of pPla-cured bacteria was on average ~1,000 fold lower than that of the wild type, confirming that pPla is required for achievement of high *Y*. *pestis* loads in the bubo. Furthermore, in the presence of pPla mean *Y*. *pseudotuberculosis* bacterial titers increased significantly to reach levels similar to those of Yp.wt (Fig 3). Thus pPla, whether in *Y*. *pestis* or a *Y*. *pseudotuberculosis*, enhances bacterial expansion in the dLN.

Since Pla is a multifunctional protein, we wanted to determine whether its capacity to promote bacterial multiplication in the dLN was due to its proteolytic activity. We constructed strain Yp(PlaD206A) (Table 1), which differs from Yp.wt by a single point mutation that was previously shown to abolish the proteolytic action of Pla [44]. In an *E*. *coli* expression system [44] as well as in *Y*. *pestis* [25], the D206A Pla mutation rendered the bacteria unable to activate plasminogen in vitro. We confirmed here that the D206A mutation in Pla abolishes its plasminogen activator activity (S2 Fig). Upon id injection of Yp(PlaD206A), the dLN bacterial burden of the mutant strain was strongly decreased, to a level similar to that of the pPla-cured derivative (Fig 3). To ensure that this impaired growth in the dLN was caused by the mutation, a Km-labeled pPla plasmid carrying the wild type *pla* allele was introduced into strain Yp(PlaD206A). The bacterial load of the complemented strain Yp(PlaD206A)(pPlaKm) reached levels comparable to those of the wild type *Y*. *pestis* strain (Fig 3). Therefore, the D206A mutation alone had the same impact on the capacity of the bacteria to survive and multiply in the draining lymph node as that of loss of the whole plasmid, highlighting the prominent role of the Pla catalytic activity for bacterial expansion in the dLN during bubonic plague.

### Mouse mortality correlates with dLN bacterial loads, but not with bacterial distribution and tissue damage

**Fig 4:**
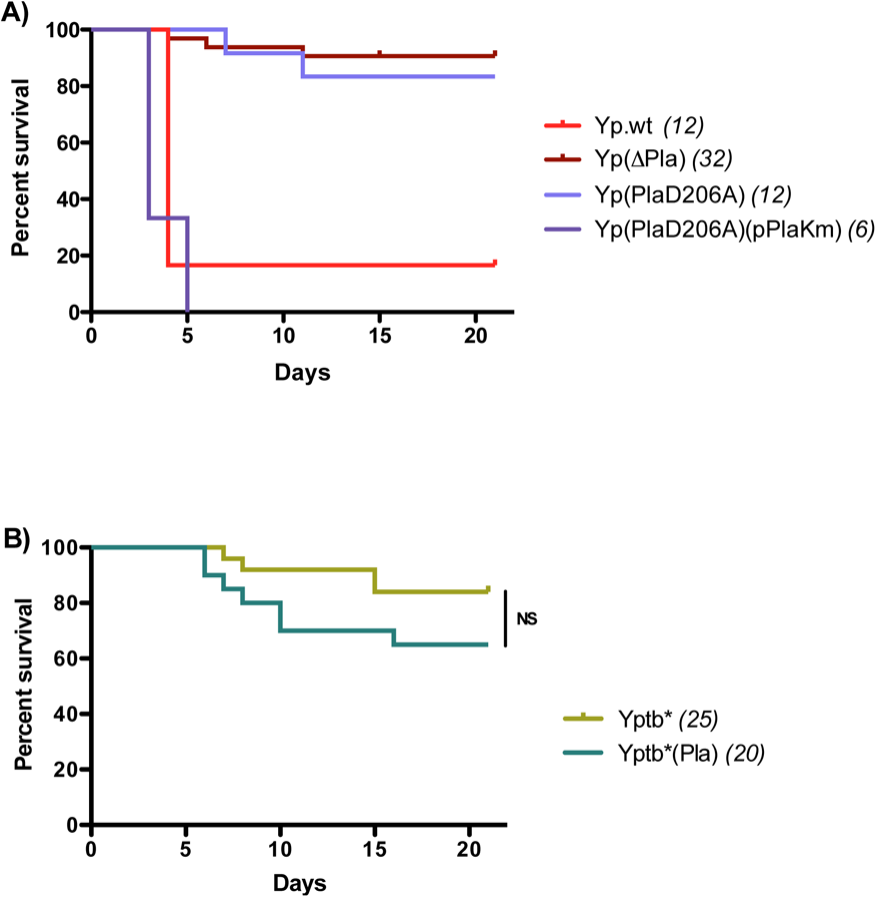
Mortality rates and kinetics in mice infected with A) *Y*. *pestis* or B) *Y*. *pseudotuberculosis* strains. Mice were infected id in the ear with 5x10^3^ cfu and followed up for survival studies. In parenthesis are indicated the numbers of animals analyzed for each strain. NS: *P* > 0.05.

Pla is a major virulence factor of various *Y*. *pestis* strains upon id or sc infections [14,15,23,24,51]. However, some strains, such as Pestoides F and strain 358, do not require pPla for full virulence [15,52]. In this study we used *Y*. *pestis* strain 6/69 because a pPla-cured derivative was available in our strain collection. To evaluate the contribution of pPla to the pathogenic potential of strain 6/69, mice were infected id with 5,000 cfu of Yp.wt or Yp(∆Pla) and monitored for 21 days after injection. The survival curves showed that the high virulence of the parental strain, which killed 83% of mice within 4 days, was strongly attenuated in the absence of pPla, with most of the animals being still alive (Fig 4A) and apparently in good health at the end of the observation period. This confirms that Pla is a major virulence factor also of *Y*. *pestis* 6/69. When the D206A mutation was introduced into *pla*, the Yp(PlaD206A) resulting strain was as attenuated as the Yp(∆Pla) derivative following injection in the ear pinna (Fig 4A). Reintroduction of a pPla plasmid carrying a functional *pla* gene restored the pathogenicity of the mutant strain (Fig 4A). Thus, in a bubonic plague model, host mortality strongly depends on the proteolytic activity of Pla. Furthermore, since we showed in this study that Pla is not required for *Y*. *pestis* 6/69 to produce large-scale tissue damage and bacterial infiltrating pattern, our data demonstrate that histopathological lesions and outcome are dissociated during bubonic plague.

In contrast, production of heavy bacterial loads in the dLN correlates to the death of the animals, consistent with the possibility that reaching a sufficient level of bacterial infection in the proximal LN is a decisive step for plague pathogenicity. To further explore the pathophysiological repercussions of high bacterial titers in the dLN, we took advantage of the fact that pPla was able to boost Yptb* expansion in the dLN to levels as high as those achieved by Yp.wt, and we asked whether these increased bacterial titers would result in an enhanced virulence of the complemented strain. Accordingly, mice were infected id with Yptb*(Pla) and followed up for survival. The pPla-complemented strain killed more mice and more rapidly than did the parent strain, but the difference failed to reach statistical significance (Fig 4B). The fact that Yptb*(Pla) was not as virulent as Yp.wt although the two strains reached similar bacterial loads in the dLN prompted us to explore the ability of the two strains to disseminate beyond the dLN. Spleen cfu titers at 48h did not significantly differ between Yp.wt and Yptb*(Pla) (log_10_ medians = 6.1 and 5.3, respectively, p = 0.0952). Therefore, neither high bacterial loads in the dLN nor the ability to disseminate to the bloodstream are sufficient to cause a fatal outcome. Other *Y*. *pestis*-specific pathophysiological mechanisms must play a role during bubonic plague to promote efficient host killing.

### Pla proteolytic activity protects *Y*. *pestis* from massive destruction in the dLN

**Fig 5:**
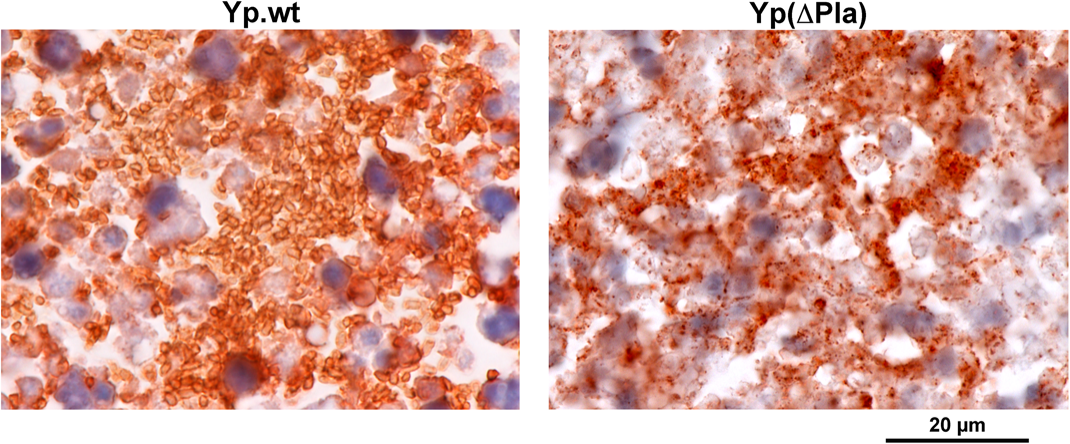
Impact of pPla on bacterial morphology in the dLN. LNs were collected 48h after id inoculation of 5x10^3^ cfu of the indicated strain and sections were immunolabeled with an anti-*Y*. *pestis* antiserum. In the left panel, the orange-brown staining delineates abundant and apparently intact bacteria typical of wild-type *Y*. *pestis* (Yp.wt) infections, contrasting with the punctate staining pattern observed after infection with the *Y*. *pestis* variant devoid of pPla (Yp(∆Pla), right panel).

Pla-mediated bacterial expansion in the LN at 48h may result from: (i) a higher capacity to reach the dLN, (ii) an increased multiplication rate and/or iii) a lower death rate of the bacteria in the organ. Cfu enumerations of Pla-negative and wild type *Y*. *pestis* bacteria at an early time point (24h) pi showed that they were present in similar amounts in the dLN (S3 Fig), indicating that pPla is not required for the initial colonization of the dLN and that the difference in bacterial loads observed at 48h pi results from mechanisms that take place after translocation to and multiplication in the LN. High magnification images of 48h dLN preparations immunostained to highlight bacteria revealed differences between wild type and Pla-deficient *Y*. *pestis* infections that were not visible at lower scale. In dLNs infected with Yp.wt, large clusters of bacteria with an intact shape were visible (Fig 5). In contrast, sections of dLN infected with Yp(∆Pla) displayed a fragmented staining pattern, made of irregular dots suggestive of bacterial debris (Fig 5). Similar observations were made on Yp(PlaD206A)-infected dLN sections (S4 Fig). A consistently low number of intact bacteria per section (0 to 12) could be seen across all (N = 26) Yp(∆Pla)- or Yp(PlaD206A)-infected dLN preparations examined. The massive difference in bacterial integrity between wild-type and Pla-deficient *Y*. *pestis* was highly consistent. No exceptions were found among all infected LN preparations examined. These results thus indicate that a major role of Pla in the dLN is to prevent bacterial destruction, presumably by counteracting the host immune system and/or overcoming nutritional limitations.

## Discussion

During the evolutionary process of its emergence from *Y*. *pseudotuberculosis*, *Y*. *pestis* acquired the multifunctional protein Pla, which is a powerful determinant of virulence and dissemination [23,25]. The best-characterized function of Pla *in vitro* is the up regulation of the host fibrinolytic system by both proteolytic transformation of the precursor plasminogen to active plasmin and inactivation of plasmin inhibitors [28,53]. Plasmin, a broad-spectrum serine protease, is the main fibrin-clot degrading enzyme and is thus central to the coagulation/fibrinolysis balance. Because its targets include procollagenases and structural proteins of interstitial matrices and basement membranes, it is also important in connective tissue homeostasis [54]. Direct or indirect activation of the host plasminogen is a common invasive strategy among pathogenic bacteria belonging to diverse genera, such as *Streptococcus*, *Staphylococcus*, *Borrelia*, *Helicobacter*, *Bacillus*, *Salmonella* and *Leptospira* [26,55–57]. This strategy was also found in parasites and fungi [58–60]. It has been suggested that a function of bacterial plasminogen activating systems is to destabilize host barriers created by fibrin and extracellular protein networks to enable bacterial expansion [53,55,61,62], in a way reminiscent of the use of plasmin by metastatic cancer cells [63]. Although there is little *in vivo* evidence to support this “bacterial metastasis” hypothesis, there have been several reports of bacterial plasminogen activators promoting the crossing of reconstituted extracellular matrices (ECM) and basal membranes *in vitro* [55,61,64]. The dramatic LN disruption observed in bubonic plague, associated with exceedingly high amounts of bacteria infiltrating the organ, led us to speculate that, within the frame of the bacterial metastasis model, these two features were pathogenetically linked.

However, using *Y*. *pestis* mutants lacking Pla or its proteolytic activity in a mouse bubonic plague model, we found that extensive tissue damage and uncontrolled bacterial burdens are uncoupled. The destructive alterations of the bubo characteristic of *Y*. *pestis* infections do not require the Pla-encoding plasmid pPla, which is nonetheless key to bacterial outgrowth in the organ. Likewise, introduction of pPla in *Y*. *pseudotuberculosis* increased the bacterial load up to wild type *Y*. *pestis* levels, but did not result in severe histological alterations of the dLN. Hence, the mode of action of Pla underlying its virulence potential is not primarily to extensively disorganize the dLN matrix protein network to clear the way for bacterial spread. Conversely, in the absence of pPla the tissue breakdown of the *Y*. *pestis*-infected dLN is not sufficient to promote bacterial accumulation.

Since Pla proteolysis is not involved, the mechanisms leading to dLN damage remain to be determined. Inflammatory responses to infections are normally accompanied by tissue destruction in and around infectious foci, chiefly owing to the release of various polymorphonuclear neutrophil (PMN) toxins, among which matrix metalloproteases and the serprocidin family of antibiotics proteins degrade most of the ECM components [65–67]. Therefore the PMN response visible in *Y*. *pestis*-infected LNs, although disorganized, could at least in part account for the observed tissue alterations. However, *Y*. *pseudotuberculosis*-infected dLNs are abundantly infiltrated by PMNs [16] without exhibiting *Y*. *pestis*-like destructive lesions. Apoptosis of immune cells, a hallmark of severe sepsis [68,69], may be another cause of the profound cell depletion of wild type *Y*. *pestis*-infected buboes, but is unlikely to play an important role during the less severe infection caused by the Pla-deficient strains.

While pPla is not the determinant of LN destructions, our observations confirm quantitatively its critical importance in the formation of bacteria-ridden buboes. We previously observed [16] and we confirm here that *Y*. *pseudotuberculosis* is significantly less abundant than *Y*. *pestis* in the infected dLNs at 48h pi. This difference in bacterial load between *Y*. *pestis* and *Y*. *pseudotuberculosis* is completely abolished when pPla is introduced into *Y*. *pseudotuberculosis*. pPla is thus a key genetic element to endow the bacteria with the capacity to survive and multiply in the dLN, and we further show that this capacity is due to the proteolytic activity of Pla. This protein could act on the bacterial load either by protecting the bacteria from the bactericidal action of innate immune defenses, or by providing them with an environment favorable for their growth. Our observation that the LNs of mice infected with *Y*. *pestis* strains lacking Pla catalytic activity contained many bacterial debris with few intact *Y*. *pestis* cells, reveals that Pla protects bacteria from undergoing lysis in the host. It is likely that most of the bacterial destruction takes place in the dLN, instead of the bacterial debris being drained from the dermis, because on day 1 pi, live Yp(∆Pla) cells are present in quantities comparable to those of wild type *Y*. *pestis*, their titers subsequently declining between 24h and 48h. Among the innate bacteriolytic factors that have been tested, Yp(∆Pla) is resistant to complement [23] but sensitive to the cationic antimicrobial peptides (CAMPs) human LL-37 and murine CRAMP [29]. CAMPs are small amphipathic molecules that bind to lipid components (hydrophobic region) and phospholipid groups (hydrophilic region) of the bacterial cell membranes, thereby causing disintegration of the lipid bilayer structure [70]. It has been reported that some CAMPs are targets of Pla and other bacterial omptins *in vitro*, and the proteolytic function of Pla prevents bacteriolysis by LL-37 and CRAMP [29,71–73]. This protection, however, was only effective in the absence of the F1 capsule, which is normally expressed in the bubo. This implies that degradation of the above CAMPs is not likely to account for the dramatically different survival rates in the bubo between wild-type and Pla-defective *Y*. *pestis* strains. Other possible mechanisms of Pla-mediated protection against *in vivo* bacteriolysis include targeting of other factors of the immune system or providing essential nutrients requiring a proteolytic degradation to become available to bacteria in the LN environment.

It is also worth noting that removal of pPla from *Y*. *pestis* or inactivation of the proteolytic activity of Pla impaired the bacterial load in the dLN to an extent higher than that of the naturally pPla-negative *Y*. *pseudotuberculosis* strain. *Y*. *pseudotuberculosis*, the ancestor of *Y*. *pestis*, is an enteropathogen that has a tropism for lymphatic tissues and in particular for the mesenteric lymph nodes during its transit through the intestinal tract. Numerous genes were either lost or inactivated in *Y*. *pestis* after it evolved from *Y*. *pseudotuberculosis* [22,74]. It is thus possible that during its evolution, *Y*. *pestis* lost some of the *Y*. *pseudotuberculosis* ancestral functions involved in bacterial survival in LNs, while acquiring pPla, which conferred a higher capacity to survive and multiply in these lymphoid organs. Interestingly, the same phenomenon was recently reported in a study involving *Y*. *pestis* Pestoides F, a pPla-negative intermediate between *Y*. *pseudotuberculosis* and modern *Y*. *pestis*. In a murine model of pneumonic plague, Pestoides F was more fit than ∆Pla-*Y*. *pestis* to colonize the lung, suggesting that Pestoides F was still harboring the *Y*. *pseudotuberculosis* ancestral functions subsequently lost during the evolution of *Y*. *pestis* [75]. Another consequence of this evolution may be the bottleneck effect that restricts access to the dLN from the dermis [76]. It is interesting that in our work and in other studies [76–78] a consistent 10% fraction of animals showed no bacteria in the dLN following id challenge with *Y*. *pestis*, while we detected bacteria in the dLN of all mice that had received *Y*. *pseudotuberculosis* cells in the ear pinna. This suggests that the bottleneck effect is linked to the loss of one or several *Y*. *pseudotuberculosis* function(s) involved in the access to lymphoid tissues, and that the acquisition of pPla did not fully restore the ability to overcome this effect.

In conclusion, this study unraveled pathophysiological relationships between components of the host response to *Y*. *pestis* infection and the role of pPla in this process. While high bacterial titers, tissue destructions and virulence were expected to be tightly linked, we show here that the extensive histological lesions observed in the bubo do not require Pla and are not associated with mortality. In contrast, bacterial loads in the dLN correlate with mortality and are Pla-dependent. However, achieving a high bacterial burden in the dLN is not sufficient for full pathogenicity. It has been proposed that Pla contribution to virulence was to catalytically break down protein barriers that would confine the bacteria and restrain their spread. However, since Pla is involved neither in the initial colonization of the organ nor in dLN destruction, this mechanism is unlikely to be essential for the development of the bubo. Our results show that achieving high bacterial burdens in the LN might be a critical step in plague pathogenesis and that one major role of Pla is to protect *Y*. *pestis* cells from the bactericidal action of the dLN environment.

## Supporting Information

S1 Fig Grouping of LN lesion patterns. Sections from infected dLNs were examined for the presence or absence of criteria of tissue alterations and inflammatory reaction as previously defined [16], and the resulting patterns were subjected to clustering analysis. In this representation, each dot represents one LN, the color code indicates the infecting strain (denoted as in Table 1) and the distance between dots inversely correlates with the degree of similarity of the histology profiles. The result shows groups of histology profiles delineated by the infecting species, but not by the presence of Pla.
(TIF)

S2 Fig Proteolytic activity of various *Y*. *pestis* derivatives. Strains (10^7^ cfu/well) were assayed for their ability to activate *in vitro* plasminogen into plasmin, as revealed by the cleavage of a chromogenic plasmin target, in the presence of plasminogen (+Plg) in the reaction mixture. The experiments were performed twice with duplicate measurements, and shown here are means and standard errors of the four values.
(PDF)

S3 Fig Early colonization of the dLN in the absence of pPla. Cfu enumerations in the dLNs were done 24h after id inoculation of ~5x10^3^ cfu of the Yp(ΔPla) strain. For comparison, data from a previous work [16] of LN colonization by wild type *Y*. *pestis* injected under similar conditions and analyzed at the same time-point are presented. Black bars correspond to the mean number of bacteria in colonized lymph nodes. The dashed line denotes the limit of detection. In parenthesis is indicated, for each group, the number of mice without detectable infection in the draining lymph node. Mean numbers of cfu in colonized lymph nodes were compared using t-test. NS: P > 0.05.
(PDF)

S4 Fig Impact of Pla proteolytic activity on bacterial morphology in the dLN. LNs were collected 48h after id inoculation of 5x10^3^ cfu of the indicated strain. Immunolabeling of sections with an anti-*Y*. *pestis* antiserum was revealed by a chromogenic reaction (orange-brown). Yp.wt, wild-type *Y*. *pestis*; Yp(PlaD206A), *Y*. *pestis* variant devoid of Pla proteolytic activity.
(TIF)

S1 Table Primers used for PCR amplification. (XLS)
