# Supplementary figures and images for: Dissociation of Tissue Destruction and Bacterial Expansion during Bubonic Plague
Source: PLoS Pathog. 2015 Oct 20;11(10):e1005222. doi: 10.1371/journal.ppat.1005222 (PMC4615631; doi:10.1371/journal.ppat.1005222)

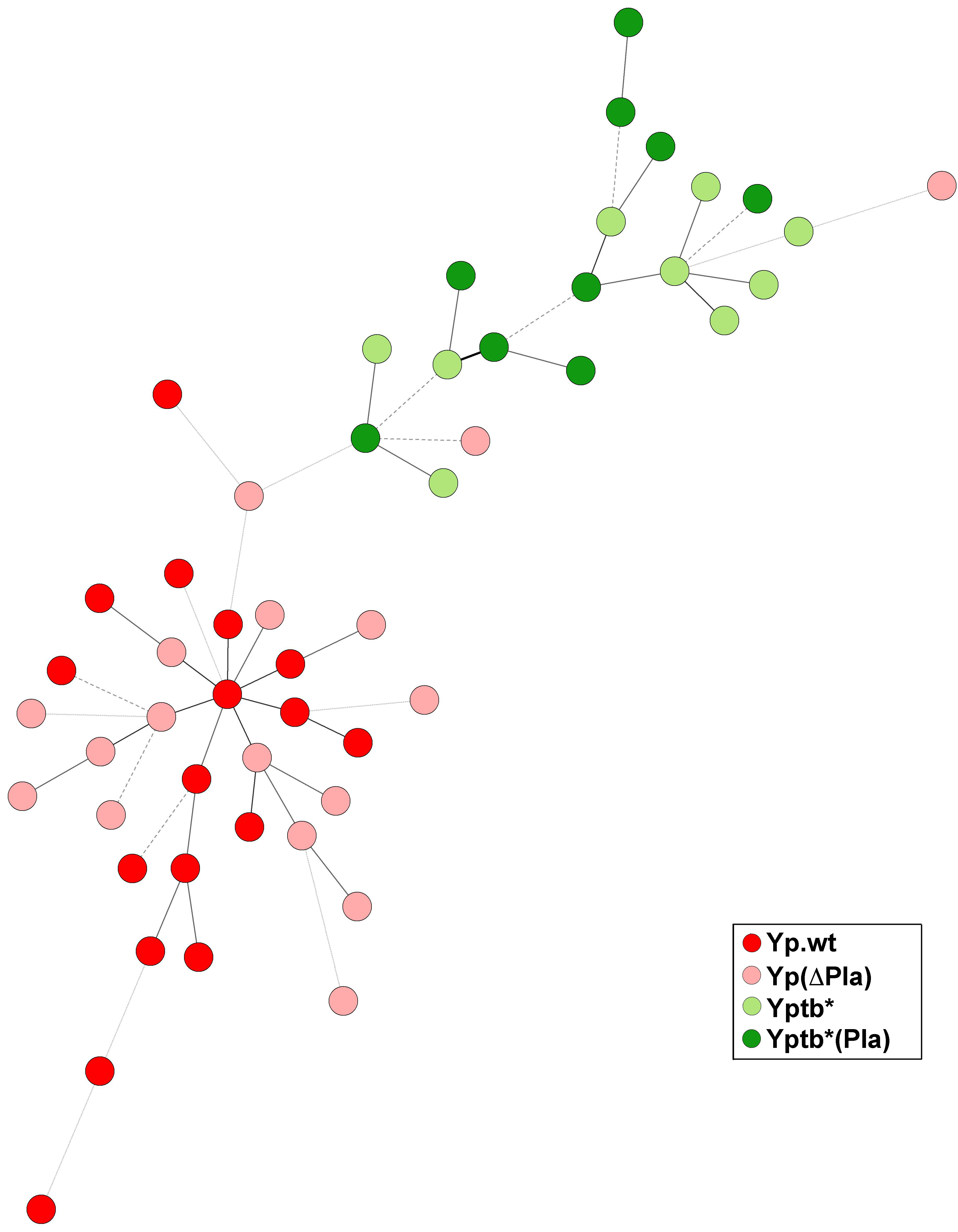

Supplement: S1 Fig — Sections from infected dLNs were examined for the presence or absence of criteria of tissue alterations and inflammatory reaction as previously defined [16], and the resulting patterns were subjected to clustering analysis. In this representation, each dot represents one LN, the color code indicates the infecting strain (denoted as in Table 1) and the distance between dots inversely correlates with the degree of similarity of the histology profiles. The result shows groups of histology profiles delineated by the infecting species, but not by the presence of Pla. (TIF) [file ppat.1005222.s001.tif]

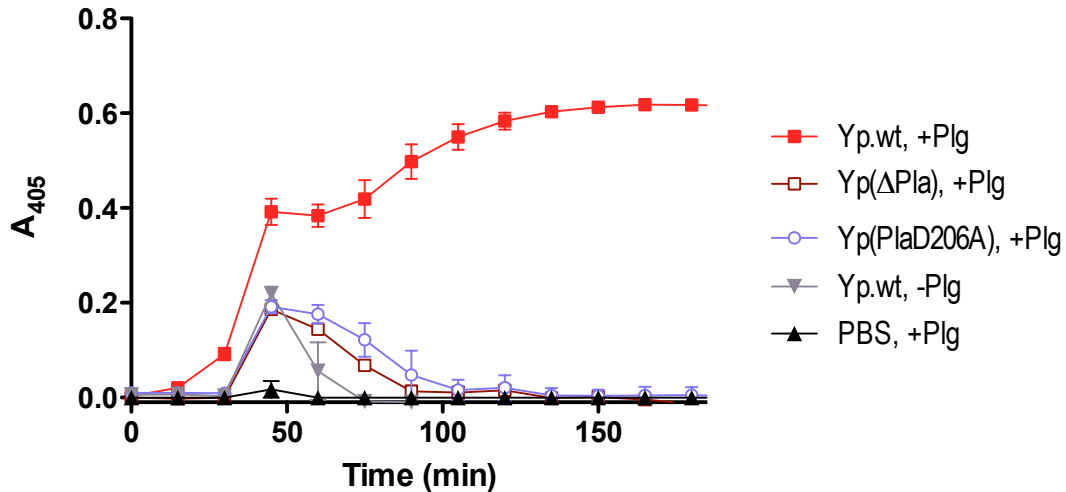

Supplement: S2 Fig — Strains (107 cfu/well) were assayed for their ability to activate in vitro plasminogen into plasmin, as revealed by the cleavage of a chromogenic plasmin target, in the presence of plasminogen (+Plg) in the reaction mixture. The experiments were performed twice with duplicate measurements, and shown here are means and standard errors of the four values. (PDF) [file ppat.1005222.s002.pdf]

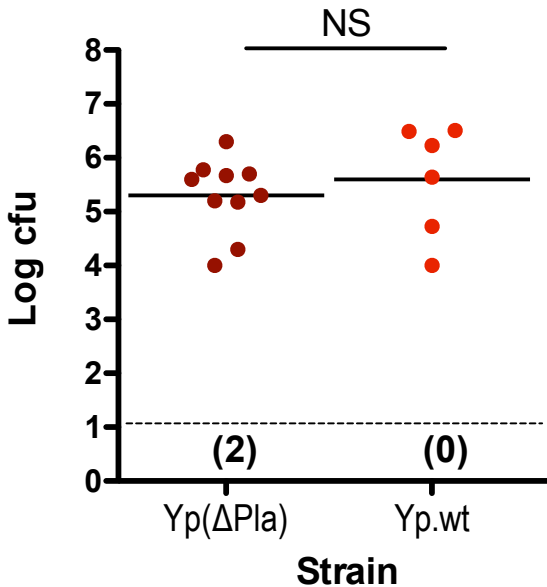

Supplement: S3 Fig — Cfu enumerations in the dLNs were done 24h after id inoculation of ~5x10^3^ cfu of the Yp(ΔPla) strain. For comparison, data from a previous work [16] of LN colonization by wild type Y. pestis injected under similar conditions and analyzed at the same time-point are presented. Black bars correspond to the mean number of bacteria in colonized lymph nodes. The dashed line denotes the limit of detection. In parenthesis is indicated, for each group, the number of mice without detectable infection in the draining lymph node. Mean numbers of cfu in colonized lymph nodes were compared using t-test. NS: P > 0.05. (PDF) [file ppat.1005222.s003.pdf]

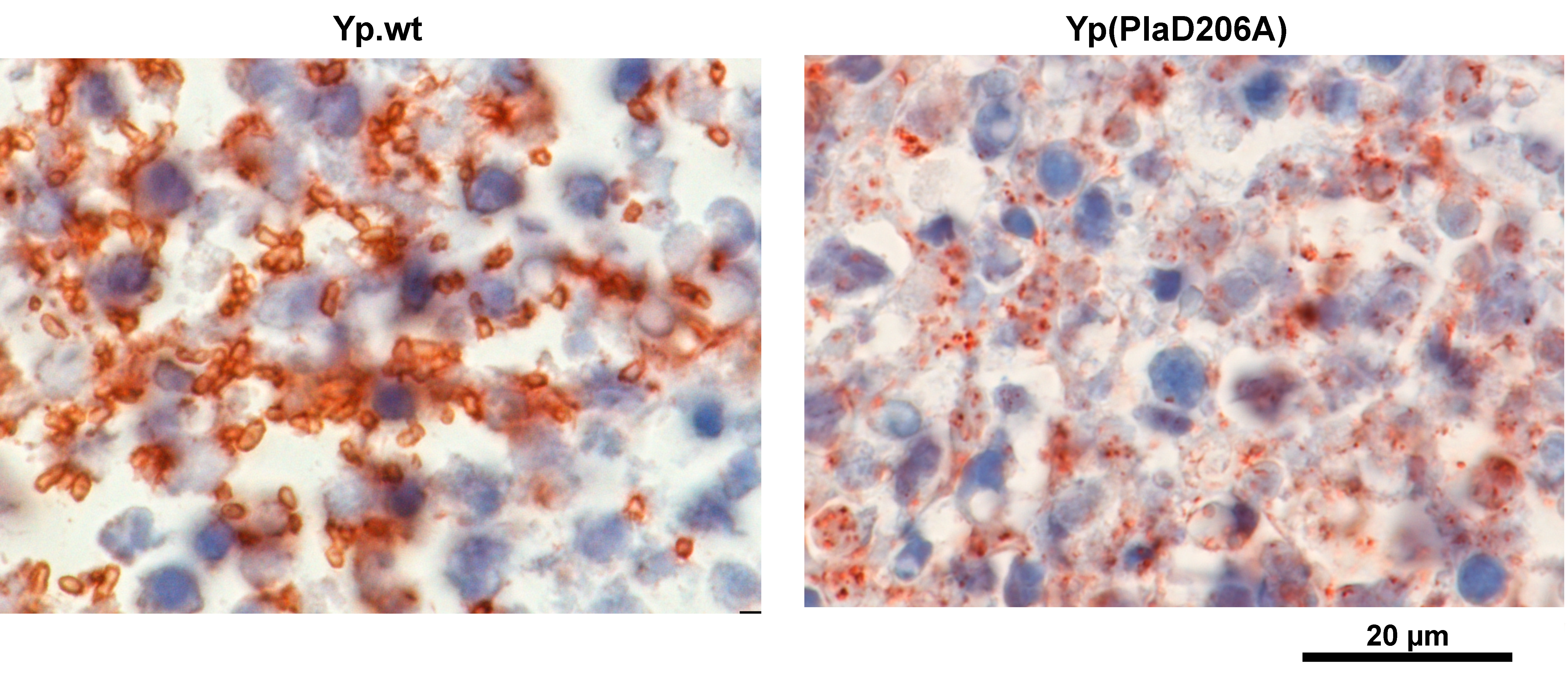

Supplement: S4 Fig — LNs were collected 48h after id inoculation of 5x10^3^ cfu of the indicated strain. Immunolabeling of sections with an anti-Y. pestis antiserum was revealed by a chromogenic reaction (orange-brown). Yp.wt, wild-type Y. pestis; Yp(PlaD206A), Y. pestis variant devoid of Pla proteolytic activity. (TIF) [file ppat.1005222.s004.tif]
